# Characteristics of Health-related Text Messages Preferred by Medically Underserved African-American Patients with Diabetes

**DOI:** 10.7759/cureus.5743

**Published:** 2019-09-24

**Authors:** Aniekan N Udoko, Joyce Graff, Samantha Ransone, Mace Coday, Justin D Gatwood, James E Bailey

**Affiliations:** 1 Pediatrics, University of Tennessee Health Science Center, Memphis, USA; 2 Pediatrics, St. Jude Children's Research Hospital, Memphis, USA; 3 Preventive Medicine, University of Tennessee Health Science Center, Memphis, USA; 4 Pharmacy, University of Tennessee Health Science Center, Nashville, USA; 5 Internal Medicine, University of Tennessee Health Science Center, Memphis, USA

**Keywords:** text messaging, diabetes, self-management, african americans, medically underserved areas, qualitative research, focus groups, primary care, culturally appropriate technology

## Abstract

Introduction

Text messaging (TM) is increasingly used by the U.S. medical practices and healthcare delivery systems, but little is known about preferences of medically underserved minority patients for TM supporting improved self-care decisions. We sought to determine the characteristics of text messages and TM programs preferred by African-American patients with diabetes in medically underserved areas.

Methods

This convergent mixed methods study employed a self-administered survey and focus group interviews. Quantitative and qualitative data were collected simultaneously, analyzed separately, and merged to provide a holistic view of the TM characteristics patients preferred. Participants (N = 36) were recruited from a medically underserved area in Memphis, Tennessee. Focus group data were uploaded into the NVivo qualitative data analysis software program, and main themes were identified. Standard frequencies were calculated for survey responses.

Results

Participants ranged in the age of 22-74 years (M = 54.1; SD = 14.6) were predominantly female (77.8%), African-Americans (88.9%), and had at least a high school education (91.7%). A majority used mobile phones for sending (69.4%) and receiving (72.2%) text messages. Participants wanted to receive daily (44.4%) or weekly (47.2%) text messages from their healthcare provider (61.1%), or a motivational message program (33.3%). They preferred actionable messages with a positive tone and wanted options to customize message type, content, and frequency according to their preferences, goals, and needs.

Discussion

Medically underserved African-American diabetes patients want customized text messages that are practical, actionable, encouraging, and from their doctor. Healthcare providers seeking to develop patient-centered TM programs for medically underserved minority patients should personalize and tailor messages according to patient preferences, health goals, and self-care needs.

## Introduction

African-Americans are nearly twice as likely to be diagnosed with diabetes as Whites and more likely to suffer from complications associated with diabetes [1]. African-Americans are also more likely to live in medically underserved areas with inadequate primary care and support for effective diabetes self-care [2-4]. Nationwide, only 43% of all patients with diabetes, 50% with hypertension, and 80% with hyperlipidemia have reached their recommended goals for average blood sugar, blood pressure, and cholesterol, respectively [5-6]. Control rates are even lower in medically underserved African-American communities, resulting in higher rates of hospitalization, complications, and death [4,7].

Health-related text messaging (TM) is increasingly being used in practices and health systems across the country to engage patients with chronic disease in self-care [8]. Research suggests that strategies aimed at incorporating self-care interventions into the primary care infrastructure can significantly improve the management of the chronic disease [9-11]. Preliminary research suggests that TM may be a particularly low-cost and effective approach for supporting self-care in primary care settings [12-16]. Furthermore, TM is practical, widely available and is less costly than other electronic forms of physician-patient communication [12].

However, despite the growing interest in determining TM efficacy and effective best practices in primary care, little is known about patient preferences for TM program characteristics. Previous work in this area is preliminary at best, and although studies have assessed interest, acceptance, and effectiveness of a TM program, the patient perspectives on the types of TM they desire is lacking [12,14,16]. Even less is known about the perspectives of medically underserved health-seeking populations. Humble and colleagues demonstrated that African-American diabetic patients in medically underserved areas are very interested in receiving text messages from their doctor’s office and were particularly interested in lab results, explanations of results, and diabetes information [12]. However, evidence regarding the most effective TM frequency, content, length, and level of interactivity are lacking.

This study combined quantitative and qualitative assessment of patient perspectives on the characteristics of text messages and primary care-based TM programs. We sought to determine those characteristics most likely to be effective in supporting improved self-care decisions by medically underserved African-Americans with diabetes. The goal of the qualitative component was to obtain more in-depth information from patients on their preferences regarding TM. We hypothesized that participants will express interest in receiving individualized motivational text messages from health providers about managing diabetes and they will have preferences for the TM program characteristics.

## Materials and methods

Methodological framework

Quantitative data were collected using a self-administered survey, while qualitative data were collected using focus group interviews. Survey and interview questions were designed to assess patient interest in a motivational TM program, as well as the types of messages they preferred. 

The pragmatist paradigm provided the framework for this mixed-methods study, which employed a convergent design [17]. This design involves the concurrent collection and independent analysis of quantitative and qualitative data using the analytic approaches best suited to the research questions and supporting the assumptions underlying the quantitative and qualitative approaches. The results of these separate analyses are merged and compared. The pluralistic nature of pragmatism supports the concurrent collection, independent analysis, and merging of quantitative and qualitative data in this study. In the practice-oriented pragmatist paradigm, the focus is on research questions, problems to be addressed, and what will work best in clinical settings [18]. Therefore, using parallel collection and analysis of quantitative and qualitative data and subsequent merging of data sets, a more holistic and real-world view can be obtained of the potential TM characteristics most likely to be effective [19-20].

Recruitment and participants

Participants were recruited from the medically underserved and majority (96.0%) African-American Memphis suburb of Whitehaven, TN through the Methodist Le Bonheur Healthcare South Hospital and its affiliated clinics. Median household income of the study population is $37,016 with approximately 28% living beyond the poverty level [8]. Potential participants included adult patients with diabetes and their family members. Level of diabetes control through average blood sugar (A1c) or self-report was not assessed or considered as inclusion criteria. Recruitment strategies included distribution of flyers at clinics, letters to patients, and telephone calls. All participants were at least 18 years of age, spoke English, and were capable of answering survey questions and participating in a focus group interview. The same participants (N = 36) participated in both survey and focus group data collection. Study participants indicated their consent by answering text message survey questions and taking part in focus group discussions. The study, TM survey, and focus group interview guide were all approved by The University of Tennessee Health Science Center Institutional Review Board.

Survey and focus group interview guide development

The TM survey and focus group questions were developed by the research team using standardized questions and data from peer-reviewed articles [12,14,16-17]. Qualitative, quantitative, and medication adherence research experts assisted in refining the survey and interview guide to ensure accuracy and stringency.

**Figure 1:**
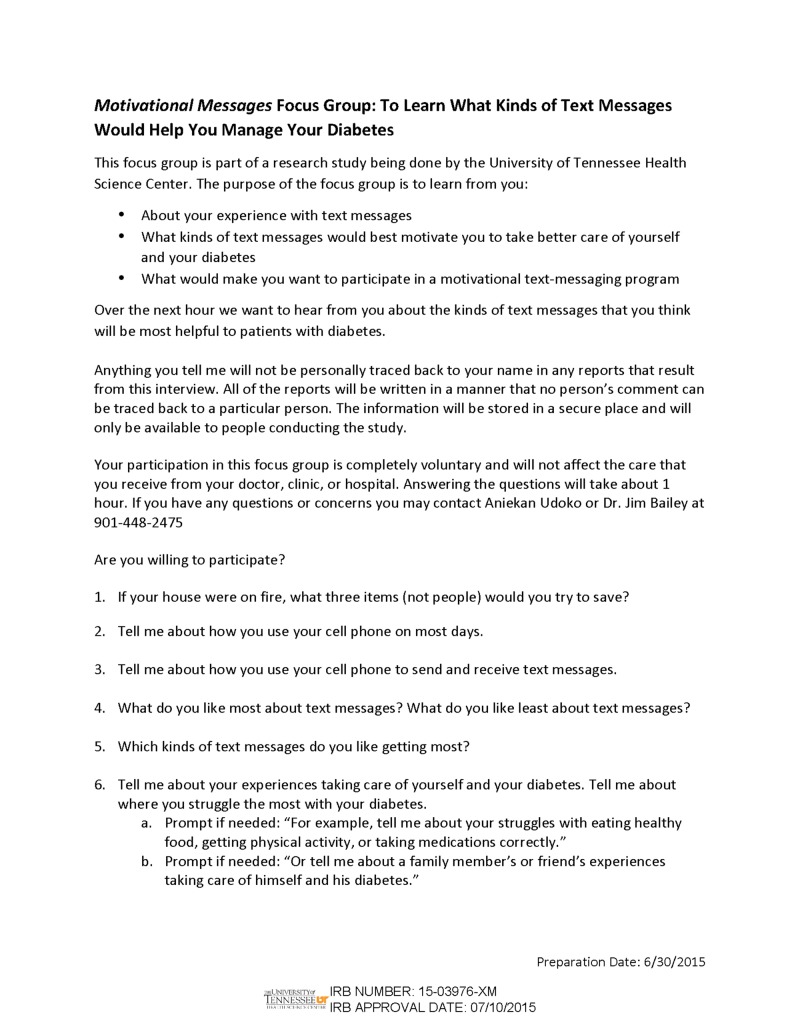
Focus group guide - Page 1

**Figure 2:**
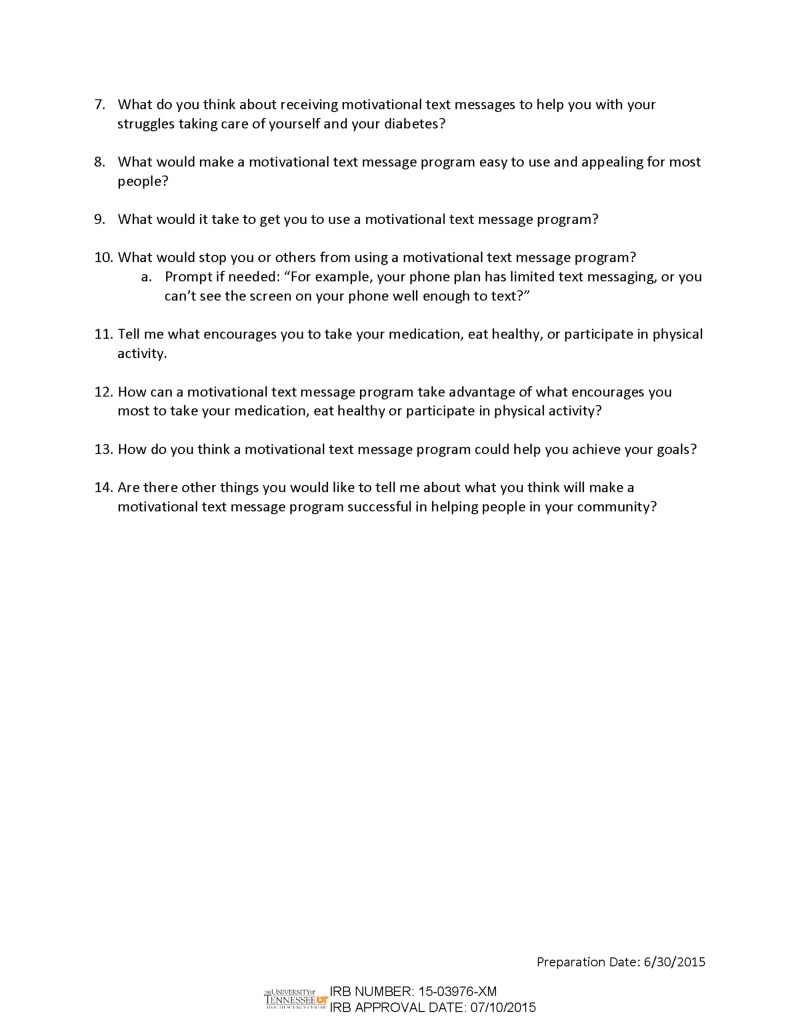
Focus group guide - Page 2

**Figure 3:**
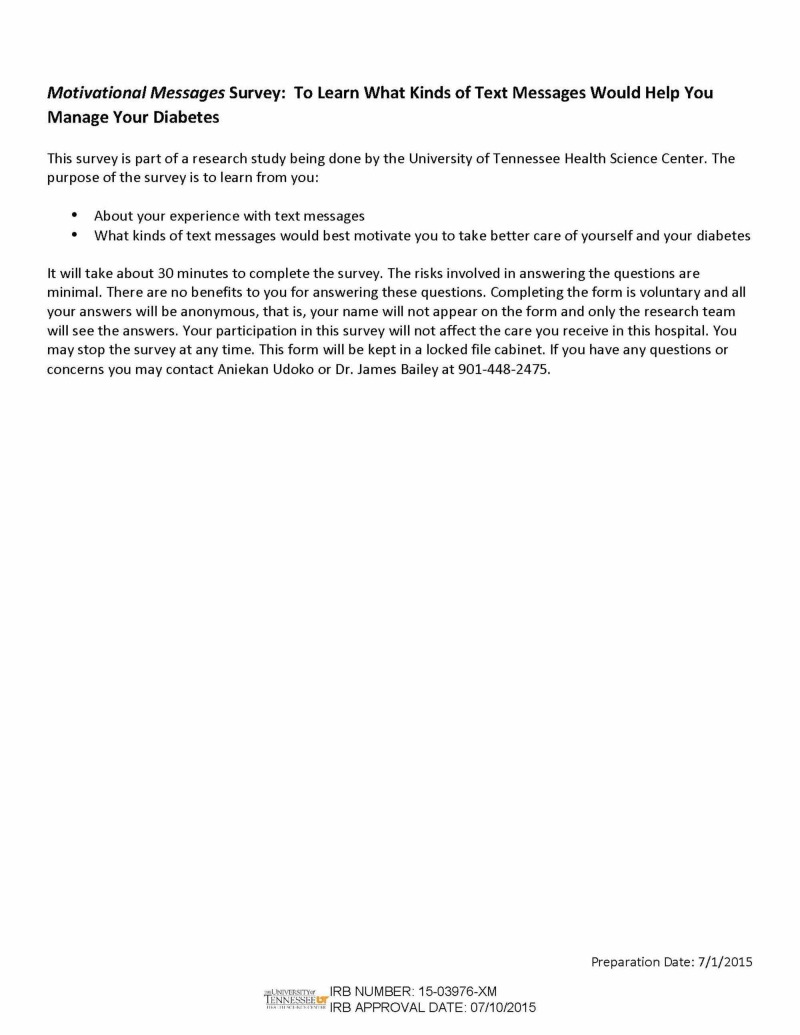
Survey instrument - Page 1

**Figure 4:**
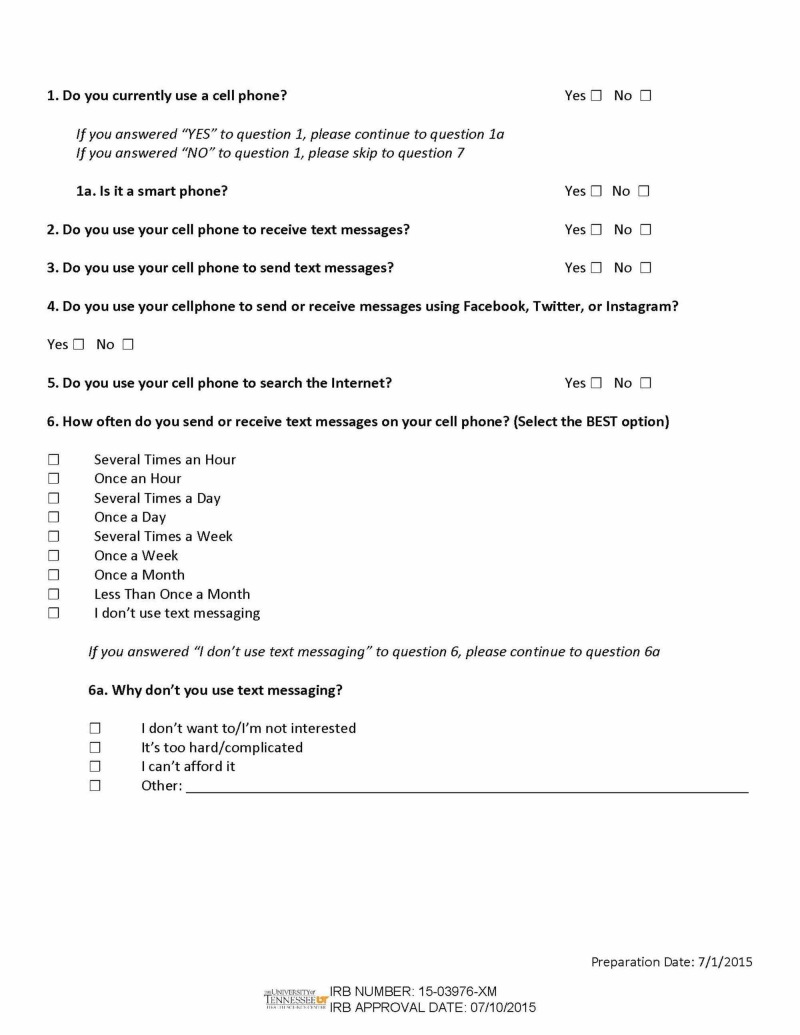
Survey instrument - Page 2

**Figure 5:**
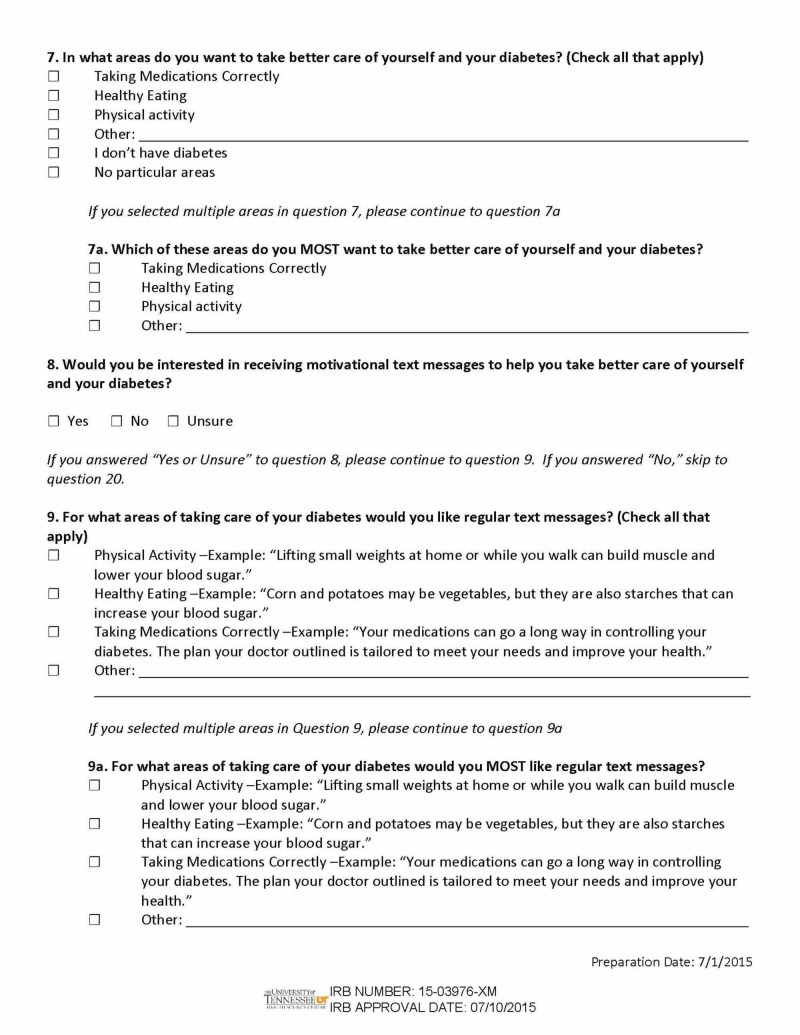
Survey instrument - Page 3

**Figure 6:**
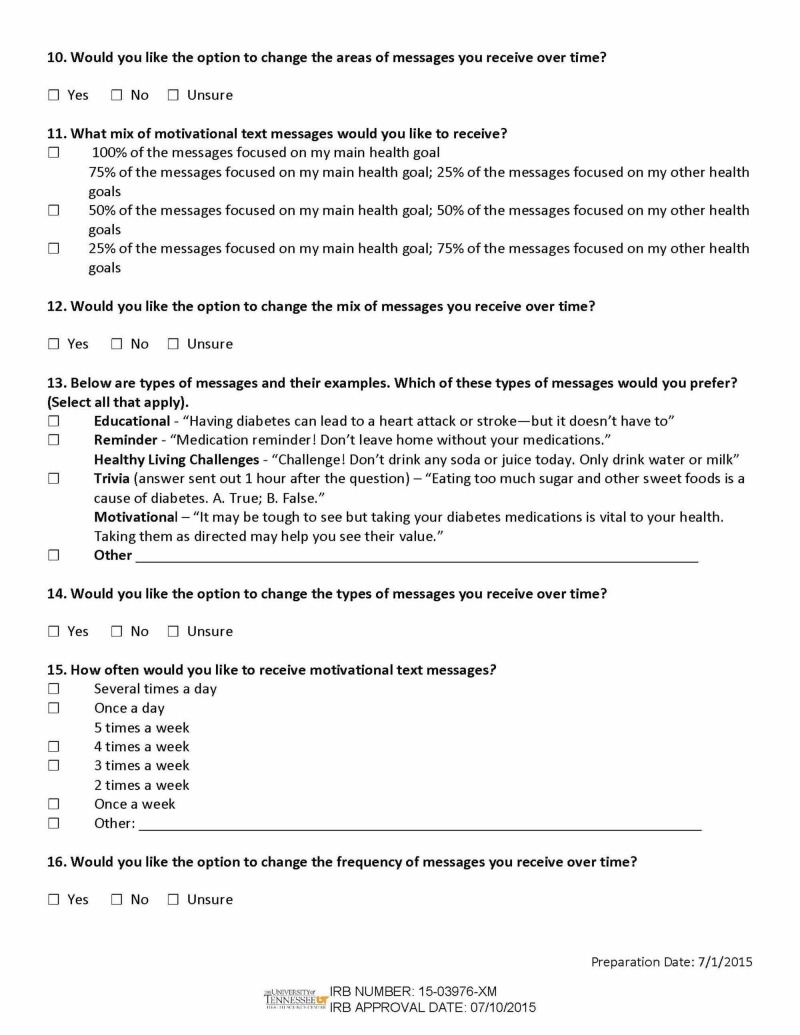
Survey instrument - Page 4

**Figure 7:**
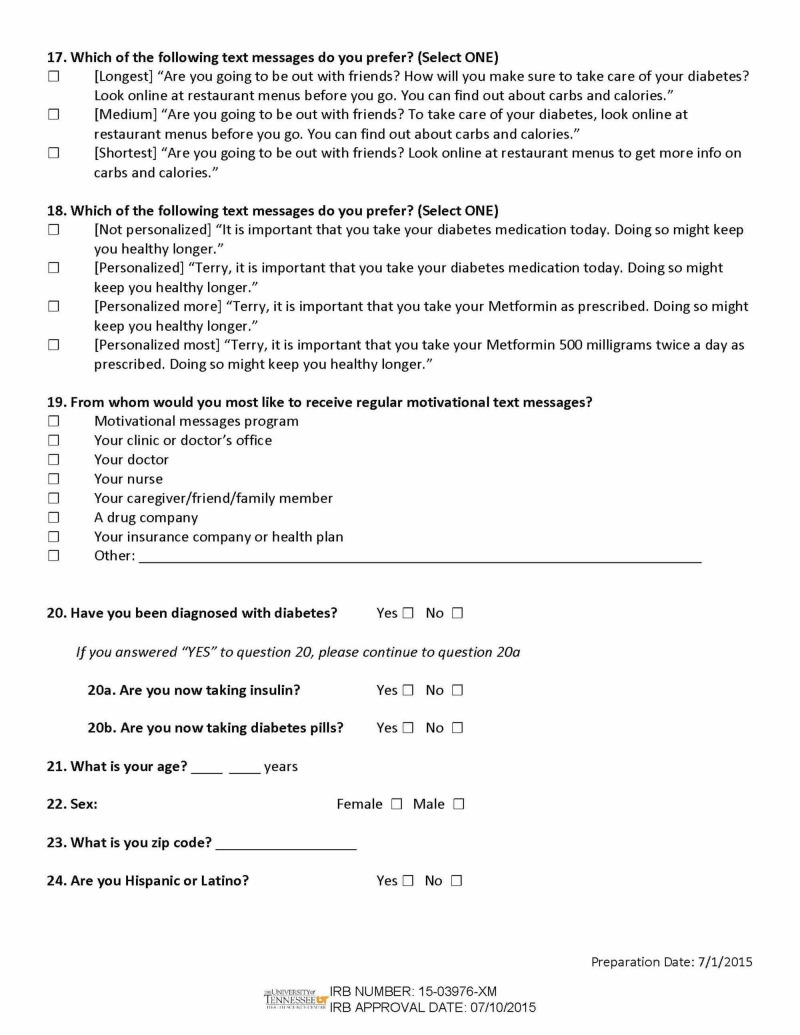
Survey instrument - Page 5

The TM survey was designed to gather demographic characteristics, data about participant technology use, and interest in a TM program supporting diabetes self-care. Health literacy was assessed using Chew’s validated one-question screening instrument and employed a 5-point Likert Scale [21-22]. The survey had 27 questions in four categories: literacy (1), demographics (7), mobile phone use and texting (6), and interest in specific health topics or types of text messages [13]. The survey (Appendices, Figures 1-2) included sample text messages to assess the preferred message topic, type, length, and degree of personalization. The focus group interview guide (Appendices, Figures 3-7) was designed to collect information about participant experiences in diabetes self-care management and text message preferences. 

Survey administration and focus group interviews

Four focus groups were conducted between June 2015 and April 2016 in hospital and clinic conference rooms at Methodist Le Bonheur Healthcare South Hospital and Christ Community Health Services. An experienced qualitative researcher trained the focus group facilitators and assistants using the guide by Krueger and Casey [23]. A medical student conducted the first three focus groups, and a faculty member facilitated the fourth one. The facilitator explained the purpose and scope of the study. Participants then completed the 27-question self-administered paper-and-pencil survey. Survey completion took about 10-15 minutes, and the focus group immediately followed. Focus group sessions were audio-recorded and lasted 90, 40, 40, and 75 minutes, respectively.

The initial focus group was conducted with members of the Diabetes Wellness and Prevention Coalition Patient Advisory Council (PAC), a patient advocacy group. Members of the PAC were newly diagnosed and chronic diabetic patients, caregivers of diabetic patients, and community members. The PAC focus group served as the pilot. The second focus group consisted of diabetic hospital employees and caregivers of diabetic patients, while the third and fourth focus group participants were diabetic patients. 

Data analysis

Survey results were analyzed using descriptive statistics. Focus group recordings were transcribed verbatim, de-identified to protect participant confidentiality, and uploaded into NVivo 11 qualitative data analysis software (QSR International, Melbourne, Australia). Two study researchers read and re-read the transcripts and used thematic analysis to identify common categories and themes [24]. Researchers integrated the quantitative and qualitative data using a joint display to visually represent and integrate of survey responses with corresponding focus group transcriptions by content area [17-18].

## Results

All participants (N = 36) completed both the survey and participated in one of the four focus groups. Participant demographics are reported in Table 1. 

**Table 1:** Participant demographics ^a^ Race and ethnicity are reported separately according to U.S. Census Bureau standards. ^b^ Percentages do not total 100% because participants could choose more than one answer. ^c^ Responses from participants who reported not sending or receiving text messages (n = 10).

Characteristics		N = 36
Age in years, median (range)		58 (22-74)
Female gender, n (%)		28 (77.8)
Black or African American race, n (%)a		32 (88.9)
Hispanic or Latino ethnicity, n (%)^a^		2 (5.6)
Education, n (%)^b^		
	Grade 1 - 11	3 (8.3)
	High school graduate	33 (91.7)
	Grade 12 or General Education Diploma	9 (25.0)
	College 1 to 3 years	13 (36.1)
	College 4 years or more	11 (30.6)
Low health literacy, n (%)		7 (19.5)
Self-care improvement areas of interest, n (%)^b^		
	Healthy eating	28 (77.8)
	Physical activity	27 (75.0)
	Taking medications correctly	20 (55.6)
	Other	7 (19.4)
Mobile phone use, n (%)^b^		
	Has mobile phone	34 (94.4)
	Has smartphone	17 (47.2)
	Sends text messages	25 (69.4)
	Receives text messages	26 (72.2)
	Uses phone to search Internet	16 (44.4)
	Uses phone to send/receive messages from Facebook, Twitter, or Instagram	0 (0)
Text message frequency, n (%)		
	Daily	18 (50.0)
	Weekly	7 (19.4)
	Monthly	1 (2.8)
	Doesn’t ever text	10 (27.8)
Reasons for not using text messaging, n (%)^c^		
	Too hard/complicated	4 (40.0)
	Don’t want to/not interested	3 (30.0)
	Don’t know how/have old phone	1 (10.0)
	Affordability	1 (10.0)
	Legally blind	1 (10.0)

Quantitative findings

Results from the descriptive statistical analysis of the TM survey are presented in Tables 1 and 2. Participants wanted to improve their self-care in the areas of healthy eating (77.8%), physical activity (75.0%), and taking medications correctly (55.6%). The majority (94.4%) reported using a mobile phone, with 47.2% using a smartphone. Participants’ use of mobile phones to send and receive text messages was similar (69.4% and 72.2%, respectively). The frequency of TM ranged from daily (50.0%) to weekly (19.4%) and monthly (2.8%). Ten individuals did not use their mobile phones for TM. 

**Table 2:** Participant preferences for text messaging program ^a^ Percentages may not total 100% because participants could choose more than one option for some questions. ^b^ See Appendix, Figures 3-7 for survey with sample messages.

Source of message			N (%)^a^
		Motivational message program	12 (33.3)
		Clinic/doctor’s office	10 (27.8)
		Your doctor	9 (25.0)
		Your nurse	3 (8.3)
		Caregiver/friend/family member	3 (8.3)
		Health plan/insurance company	1 (2.8)
		None/no response	2 (5.6)
Content			
	Topic most preferred, n (%)^b^		
		Healthy eating	27 (75.0)
		Physical activity	19 (52.8)
		Taking medications correctly	18 (50.0)
	Type or characteristics, n (%)^b^		
		Educational	22 (61.1)
		Trivia	17 (47.2)
		Motivational	7 (19.4)
		Healthy living challenges	4 (11.1)
		Reminder	0 (0)
	Personalization, n (%)^b^		
		Personalized	7 (19.4)
		Personalized more	7 (19.4)
		Personalized most	7 (19.4)
		Not personalized	12 (33.3)
		No answer	3 (8.3)
Frequency and length			
	Frequency, n (%)		
		Daily	16 (44.4)
		Weekly	17 (47.2)
		No answer	4(11.1)
	Message length, n (%)^b^		
		Short	23 (63.9)
		Medium	5 (13.9)
		Long	4 (11.1)
		No answer	5 (13.9)
Interactivity			
	Option to change message type (characteristics) over time, n (%)		
		Yes	24 (66.7)
		No	5 (13.9)
		Unsure	3 (8.3)
		No answer	4 (11.1)
	Option to change mix of message content over time, n (%)		
		Yes	24 (66.7)
		No	5 (13.9)
		Unsure	3 (8.3)
		No answer	4 (11.1)

Participant preferences for text message delivery, content, length, and message interactivity are shown in Table 2. The majority preferred for motivational text messages to be delivered daily or weekly. Although one-third preferred messages from a TM program, almost two-thirds preferred text messages from their health provider. Educational, trivia, and motivational text messages were the most preferred types of messages. The majority of participants preferred short text messages (63.9%) and personalized text messages (58.2%). And most participants wanted options to change text message type (66.7%), content (63.9%), and frequency (66.7%) over time. Examples are shown in the Appendix.

Qualitative findings

Focus group discussions yielded six major themes: 1) diabetes self-care struggles; 2) use of mobile phones; 3) perceived advantages of TM and TM programs; 4) perceived barriers of TM and TM programs; 5) desired characteristics of TM programs; and 6) undesired characteristics of TM programs (Table 3).

**Table 3:** Comments from focus group participants

Themes and Categories		Quotes
Diabetes self care struggles		
	Discipline (exercise, food, monitoring glucose, diet, insulin, taking medications, sleep habits)	Well, here’s what I struggle with the most . . . it’s getting myself up and getting some exercise.
		I struggle the most probably eating the right things, because…it’s hard to find good healthy food, you know.
		…when I was working it was a struggle to get up and get ready for work, take my medications on time and daily...
	Lack of information	I would like to be able to have more ideas on stuff. How to prepare it and how to eat them.
	Fear related to monitoring glucose	. . . my struggle is, and I guess I'm just guilty of it. You know how people have a meter, and then, you know, you prick your finger? I have, I have never done that . . . I got a fear.
	Financial difficulties (food, health insurance, medication and equipment cost)	When you go in those stores you going to monitor how much you spend and that could be a struggle, you know, for maintaining your diabetes.
		Fruit is expensive.
		And then like buying medication. All medication are not covered. It just depends on what kind of insurance you have, and that’s a big struggle.
		When you don't have any medicine at all…I'd be stretching mine because of the cost.
		I have been for three weeks without my medicine…I really, I've been telling my doctor, I said, what am I going to do?
	Time (busyness, lack of time)	Finding the time with the kind of jobs we have, we just don’t stop and exercise.
Use of cell phone		
	Age (older adults new to texting, younger adults more likely to text)	What I’m thinking is, I know young people they do not call you at all. All they do is texts. It may be just the younger generation does it.
		But when it’s concerning [older adults] they feel like they need to learn how to text. Somebody would need to teach them how to text.
		It was a while before I started texting also . . . what made me uh, start texting was because my granddaughter she said, ‘Mama, you may be somewhere where you can’t use your phone and it would benefit you.’
	Communication (with church members, with family members)	Well, on most days I just use mine just to check on my family.
		I’m like a caregiver at heart, and I have several people that I check on, and the one’s a deacon that is in my church is about 86. I know there's a friend of mine that’s in a nursing home that’s had a stroke, and use that at least to call people just to say hi or, you know, sometimes just cheer people up. You know, sometimes people feel better when they think somebody care about them.
	Convenience (call for prescription, when away from home)	Call in for my prescriptions. Call and get my Metformin.
		I won't use mine unless I'm away from home, to check on my grandbabies and my kids, and let 'em know where am at.
Perceived advantages of text messaging and a text messaging program		
	Support from others in community (using text messaging to facilitate support groups and classes)	And try to have a group meeting about once a month.
		Have some classes. Some of them offer like free cooking classes and stuff in the church.
	Convenient (avoids long conversation, less intrusive, quicker response, respond at own discretion, straight to the point, informative, privacy)	The texting is less intrusive than a call, so people will respond quicker.
		If I call they are gonna talk too long, so I just text…because everybody has friends that just want to go on and on. And on and on.
		Straight to the point.
Perceived barriers of text messaging and a text messaging program		
	Cost	…a lot of people [may] opt out…for that reason alone. Because if you receive the text message you still have to pay for it.
	Inconvenience (bothersome, delays conclusion to conversation)	Them bothering me.
		I would just rather say what I gotta say and be through.
	Lack of community	Now, my take on that is I like groups. I like talking to people. Messages is kind of isolating. I’m already isolated in my work.
	Safety issues with texting and driving	Uh, it may be stretching it out too, also this proclivity we have to text, drive, and this thing we have about every time the phone rings we have to check it.
	Technology use (don’t or rarely text, don’t use cell phone, unfamiliarity with technology, primarily use landline)	Well, I know how to text, but I don’t text; and I very rarely use my cell phone. I’m at home most of the time. I have a land line so that’s what I use all of the time.
		Yeah. But I don't text ... But if I get or receive a text, I will call the number back.
	Visual impairment	One thing you do want to be mindful of…now for those of us who are visually impaired, uh texting is a whole different world...very rarely do you find someone that is totally blind texting people.
Desired characteristics of text messaging program		
	Customizable (alternate options to text messaging, frequency, interactivity, message content, message content based on age, message length, “opt out” and change settings option, personalization, timing)	Provide that resource for the visually impaired where they could get a voicemail or voice to text or whatever the deal.
		Not too often.
		Not more messages. If I get more than one a week that’s enough.
		Once a day.
		More than twice a day.
		But, if I had to reply back to each one of them, even though I’m going to choose when I stop to actually read them. If I had to reply back to each one it becomes a nuisance.
		I like short and to the point.
		Because your case is going to be different from my case.
		Maybe they can have a set time. You know, okay, this is the time that they going to call me. So you expect that. You know, that this is the time they going to call me.
		Not early in the morning.
	Educational (community events; diabetes complications; diet)	Free invitations for stuff around Memphis... You know, the free …[Zumba] classes.
		Some people really do not really realize what diabetes can actually do to you until it happens.
		Being a diabetic…first of all, you feel so unintelligent. You just feel like all these words: carbs, this, that, and the other, you can’t just pick up and start eating like you’ve done all of your life.
	Message type (based on age, instructional, pictorial, reminder, tone, simplicity)	‘Don’t forget to do this, don’t forget to do this.’
		Pictures…some kind of pictures. Uh, if they are the best means of conveying a message. Uh-uh somehow, they work on your brain better.
		I would like to get reminders but I would not want to respond back to each one.
		I would just want some general inspirational, you know ‘Have a great day’ kind of thing. Uh, you know some nice quote that’s just going to change your mindset that day…Rather than ‘you should do this, and you should do that’.
		And the language simple too.
Undesired characteristics of text messaging program		
	Solicitation (advertisements, unsolicited messages)	Then at the end of it is a sales pitch.
		I don’t like unsolicited messages.
	Timing (avoid middle of workday, inappropriate time or place)	I would suggest not doing it, not sending them during work hours. Because I have a tendency to overlook stuff.
	Too many messages	Mine would be harassment.
		Then only thing that would stop me is if it gets to be annoying.

Diabetes Self-care Struggles

Participants disclosed and discussed their struggles with diabetes self-care surrounding discipline, lack of education, fear related to glucose monitoring and injecting insulin, financial difficulties, and time limitations. Study participants frequently cited lack of discipline as their primary obstacle to achieving their self-care goals. In particular, they cited difficulties in finding the discipline to exercise, eat healthy foods, monitor glucose, take medication properly, and establish good sleep habits. Participants reported that they wanted to know what to eat, how much to eat, and how to prepare food. They also identified financial difficulties as an impediment to maintaining a healthy diet or adhering to medications. Participants also expressed concerns about paying for health insurance, medication, and equipment. Others said they were unaware of the exact nature of their illness and associated complications. Fear of needles was a barrier for insulin adherence. Participants suggested that a TM program could help them overcome these struggles by providing helpful reminders, as well as information about grocery shopping, food preparation, exercises that fit individual lifestyles and schedules, and local resources to help those with financial difficulties obtain healthy foods and medications.

Use of Mobile Phone

Participants said mobile phones were a convenient way to keep in contact with family members, friends, and church members. Some participants also use their mobile phones to complete errands, while others use it as a way to check in with loved ones during the day when away from home. Participants reported that younger adults are more adept at texting, while older adults are often newer to texting. One participant suggested offering an optional TM lesson for those who chose to enroll in a TM program.

Perceived Advantages of TM

Participants identified many advantages of TM and a potential TM program, including convenience, access to information, the private nature of messaging, and helpfullness in developing a supportive community. Many participants identified TM as convenient because it prevents lengthy conversations, is less intrusive than a phone call, and allows the user to respond at his/her own discretion. Furthermore, they identified a TM program as a potentially effective method to enhance diabetes patient education both for enrolled patients and for their family or friends. Participants mentioned that TM would also allow program participants to share knowledge with loved ones. Privacy was an important concern and participants agreed that a TM program would provide a level of privacy. Lastly, while most participants were able to identify advantages of TM and a potential TM program, one participant did not identify any advantages.

Perceived Barriers of TM

Participants identified several possible TM barriers: lack of texting experience, inconvenience, compromised safety, lack of community, visual impairment, and cost. Discussions revealed participants’ concern that TM could lead to bothersome messages, and could, therefore, be less convenient than other forms of communication. Several participants voiced safety concerns about texting and driving. When comparing a TM program to a support group, one participant noted that TM can be isolating. A visually impaired participant reminded the group that many diabetes patients are blind. Lastly, a potential barrier identified during group discussions was affordability, as the cost of adding TM to a mobile phone plan might deter would-be participants.

Preferred Characteristics of TM Program

Participants preferred a customizable and educational program that features uplifting, instructional, simple TM that considers the age of the recipient and incorporates images and reminders. According to participants, a customizable program should include a voicemail option for the visually impaired. Other options identified were the frequency of TM, level of interactivity, message content, message length, level of personalization, and timing of TM to meet their needs. They differed on the degree of personalization preferred, with some preferring medication reminder text messages that were highly personalized (e.g. using patient name and medication details) and others preferring generic medication reminders. Participants also wanted to have an “opt out” option and the ability to change program settings. Many desired a program that would provide information and educational opportunities about exercise, diet, and diabetes complications. They also noted that newly diagnosed diabetic patients are suddenly faced with the task of navigating a new lifestyle and often learn about complications when it is too late. In terms of the types of TM, participants desired what one participant called a “mom message,” a message with instructions and reminders. Others wanted uplifting messages or messages with pictures that would be motivating. Additionally, participants listed product solicitation, poor timing, and too many messages as potentially undesirable TM features.

Integration of Qualitative and Quantitative Data

Table 4 provides a joint display that integrates the quantitative and qualitative data and demonstrates the convergence of themes. The table highlights how the focus group interviews enabled participants to provide in-depth information on survey topics. For example, when discussing diabetes self-care struggles, focus group participants identified financial difficulties, lack of discipline and lack of education as key barriers. In contrast, the survey focused more narrowly on diabetes self-care activities related to healthy eating, physical activity, and medication use. Similarly, while survey questions about TM focused primarily on text message frequency, focus group discussions noted importance of timing (e.g. avoiding messages in the middle of the workday).

Limitations

It is possible that the sample of patients selected for this mixed-methods study were not representative of the target population of medically underserved African-American patients with uncontrolled diabetes. The level of diabetes control among participants was not assessed. However, all participants had experience living with diabetes, were African American, and lived in medically underserved areas in the Mid-South. Previous research indicates that diabetes is poorly controlled in approximately 50% of this population [5]. In addition, the study is potentially subject to Berkson's bias, since the sample was not taken from the general population, but from a subpopulation served by a healthcare delivery system. However, this healthcare delivery system serves over 70% of African-American patients in the targeted medically underserved ZIP codes.

**Table 4:** Mixed methods integration of text messaging survey and qualitative findings

Mixed methods domains	Survey findings	Qualitative findings	Mixed methods meta-inferences
Barriers to achieving self-care goals	Self-care improvement areas: Healthy eating (77.8%), Physical activity (75.0%), Taking medications correctly (55.6%).	Diabetes self-care struggles: Discipline; Lack of information; Fear related to checking blood sugar; Financial difficulties related to food, health insurance, medication, and equipment cost; Time.	Participants describe struggles related to self-care that were not included in survey questions.
Text message personalization and customization	Personalization of text message content: Personalized (58.2%), Not personalized (33.3%), No response (8.3%). Option to change message type over time: Yes (66.7%), No (13.9%), Unsure (8.3%), No answer (11.1%). Option to change mix of message content over time: Yes (66.7%), No (13.9%), Unsure (8.3%), No answer (11.1%).	Desired characteristics of text messaging program Customizable, Educational, Message type.	Most participants prefer personalized text messages that are customized to their own health goals and needs. An “opt out” option and ability to change text message program settings is desired.
Text message frequency and timing	Frequency: Daily (44.4%), Weekly (47.2%), No answer (11.1%).	Desired characteristics of text messaging program: Customizable (frequency) Undesired characteristics of text messaging program: Unsolicited messages, Avoid middle of work day.	Variability of participants’ desired frequency and timing of text messages reflects the importance of a customized text messaging program.
Text message content and tone	Topic most preferred: Healthy eating (75.0%), Physical activity (52.8%), Taking medications correctly (50.0%). Type or characteristics: Educational (61.1%), Trivia (47.2%), Motivational (19.4%), Healthy living challenges (11.1%), Reminder (0%).	Desired characteristics of text messaging program: Customizable (message content), Educational, Message type.	Personalizing text message content may remind patients of their desire to change. Diabetic patient education includes addressing newly diagnosed patients and patients’ secondary or tertiary health goals. Text messages that are positive in tone, motivational, inspirational, and incorporate images or emoticons are important.

## Discussion

Many studies have explored the use of health-related text messages to influence patient self-care decisions and health behaviors, but few studies have specifically assessed the types of messages preferred by African-American patients with chronic illness in medically underserved areas [8,13]. This study is among the first to show these patients were most interested in receiving text messages that are personalized and tailored to their interests and self-care needs. Participants’ interest in handling their disease management and improving their health outcomes is consistent with the concept of ownership of diabetes as described by Shearer and colleagues [25]. We found that a majority of the medically underserved patients in this study sample already use mobile phones for personal text messaging and are highly interested in receiving health-related text messages from their doctor’s office. Furthermore, both quantitative and qualitative findings demonstrated that medically underserved African-American patients with diabetes want support in managing their own health and believe that a TM program could help them overcome their diabetes self-care struggles.

Our study's finding that patients prefer tailored TM programs that are personalized to individual needs is consistent with research indicating that tailored TM programs are more likely to motivate patients [26-27]. For example, Horner and colleagues found that a one-size-fits-all approach led to frustration and disengagement as study participants found the messages impersonal and unrelated to their specific self-care struggles. The current study is also consistent with studies showing that patients are more likely to read messages specific to their health goals and, therefore, more likely to succeed in improving their health [26,28]. Previous studies demonstrated that personalized information can be gathered and utilized to create a TM database for each participant that is updated in real-time to reflect patients’ changing preferences over time. For example, Job and colleagues checked in with participants in a weight loss program at 12 weeks, updated their preferences and progress, and changed content, leading to increased patient accountability and engagement with the TM program [27]. Similarly, our study showed that many patients want to be able to change TM preferences over time. Future studies are needed to establish ideal levels of personalization and best approaches for incorporating patient preferences over time.

The current study found high levels of individual variability in patient preferences regarding frequency and timing of text messages, consistent with previous research suggesting that messages sent too frequently or at inconvenient times can elicit negative reactions. For example, Smith and colleagues found that adolescents who received obesity-related text messages three times a week developed negative feelings about the TM program and started avoiding or ignoring messages [26]. However, studies in other populations have shown high receptivity to daily text messages [15,29]. Recent studies that have tailored text message frequency and timing to participant preference have demonstrated high patient engagement over time [27]. Previous studies indicate that patient preferences for message timing may depend on the message type. A large and varied TM database may also aid participant engagement as objections to frequent messages may be due to boredom from similar messages or being accustomed to messages making them less effective [8,29]. Frequent check-ins and updating of participant preferences may also help negate the apathy that results from repeated messages [11,27]. 

Our study provides evidence that engaging diabetes patients in medically underserved areas could benefit from TM content being tailored to individual self-care struggles and goals. Our findings are consistent with research suggesting that text messages that are tailored to individual challenges and goals may do more to remind patients of their desire for change and improvement [26]. We found that patients may want to identify secondary or even tertiary health goals in order to be able to focus on more than one goal at a time. This is especially applicable to diabetes management as patients may struggle with more than one area of care. Personalization and the ability to change TM content is important because individual needs vary and are likely to change over time based on disease stability, progression, or symptoms. 

Lastly, the current study demonstrates that text message tone is critically important for effectively engaging African-American diabetes patients in medically underserved areas. Similar to Smith and colleagues, we found that patients disliked messages that had a negative tone or were perceived as shaming [30]. Our findings are consistent with previous studies suggesting that patients like practical and actionable messages that keep them on track and accountable [27]. In addition, the current study suggests that motivational and inspirational messages may be particularly effective in encouraging continued patient engagement, regardless of whether they relate to personal health goals. Similarly, text messages that incorporate encouraging images or emoticons [26] may provide a more natural and casual exchange, thus enhancing engagement.

## Conclusions

Primary care practices and health systems seeking to develop patient-centered TM programs for medically underserved minority patients with chronic diseases should strive to tailor health-related text messages to meet participants’ personal health needs and interests. Assessing patients’ self-care struggles, health goals, mobile phone use, perceptions of TM, and desired outcomes of the TM program during enrollment in a TM program can lead to a customized TM program that supports active participation, patient satisfaction, and responsible decision-making for better health. This study suggests that tailored TM programs may provide a patient-centered, effective, and well-received approach for supporting self-care for underserved African-American diabetic patients. Our results suggest that the most effective TM programs should have the capacity to personalize messages to meet the needs of individual participants.
